# Supplementary material for: Effect of the Addition of Different Natural Waxes on the Mechanical and Rheological Behavior of PLA—A Comparative Study
Source: Polymers (Basel). 2023 Jan 6;15(2):305. doi: 10.3390/polym15020305 (PMC9866918; doi:10.3390/polym15020305)
Supplement: Supplementary file 1 [file polymers-15-00305-s001.zip › polymers-2075400-supplementary.pdf]

polymers

Effect of the addition of different natural waxes on the mechanical and rheological behavior of PLA—A comparative study

Mónica Elvira Mendoza-Duarte [1]*, Iván Alziri Estrada-Moreno [2], Erika Ivonne López-Martínez [1] and Alejandro Vega-Rios [1,*]

[1] Centro de Investigación en Materiales Avanzados, SC, Av. Miguel de Cervantes #120, Chihuahua 31136, Mexico
[2] CONACyT-CIMAV, SC, Av. Miguel de Cervantes #120, Chihuahua 31136, Mexico
* Correspondence: M.E.M.-D. monica.mendoza@cimav.edu.mx; A.V.-R. alejandro.vega@cimav.edu.mx.

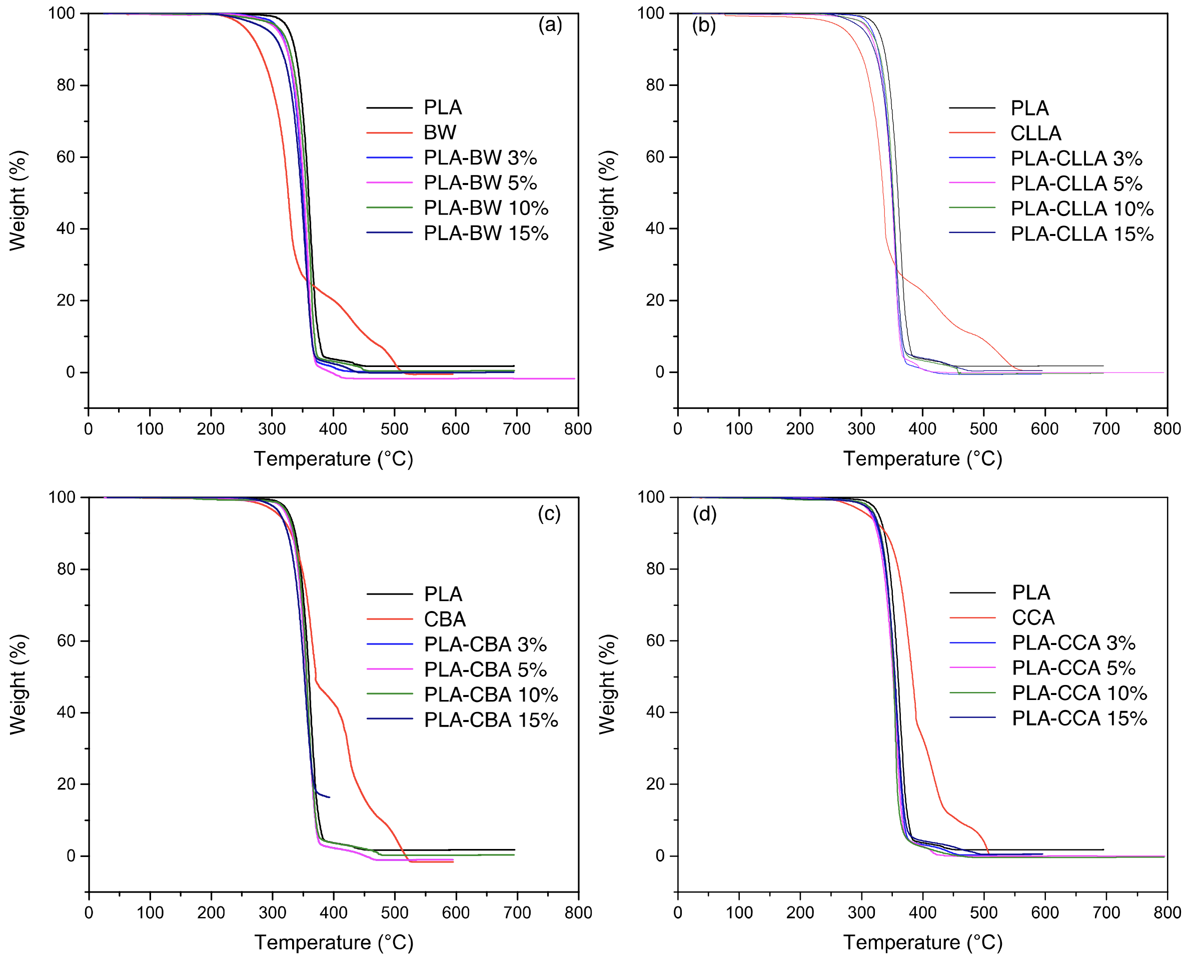

Figure S1. Thermograms of neat materials (polylactic acid (PLA), beeswax (BW), candelilla (CLLA), carnauba (CBA), cocoa (CCA)) and PLA-wax (BW, CLLA, CBA, CCA) blends. (a) PLA, BW, PLA-BW blends; (b) PLA, CLLA, PLA-CLLA blends; (c) PLA, CBA, PLA-CBA blends; (d) PLA, CCA, PLA-CCA blends.
